# Antiviral Properties of Flavonoids and Delivery Strategies

**DOI:** 10.3390/nu12092534

**Published:** 2020-08-21

**Authors:** Paolino Ninfali, Antonella Antonelli, Mauro Magnani, Emanuele Salvatore Scarpa

**Affiliations:** Department of Biomolecular Sciences, Section of Biochemistry and Biotechnologies, University of Urbino Carlo Bo, 61029 Urbino, Italy; paolo.ninfali@gmail.com (P.N.); antonella.antonelli@uniurb.it (A.A.)

**Keywords:** flavonoids, antiviral properties, viral enzymes, phytochemical delivery strategies

## Abstract

This review summarizes the latest advancements in phytochemicals as functional antiviral agents. We focused on flavonoids, like apigenin, vitexin, quercetin, rutin and naringenin, which have shown a wide range of biological effects including antiviral activities. The molecular mechanisms of their antiviral effects mainly consist in the inhibition of viral neuraminidase, proteases and DNA/RNA polymerases, as well as in the modification of various viral proteins. Mixtures of different flavonoids or combination of flavonoids with antiviral synthetic drugs provide an enhancement of their antiviral effects. Recent strategies in drug delivery significantly contribute to overcoming the low bioavailability of flavonoids. Frequent viral infections worldwide have led to the need for new effective antiviral agents, which can be identified among the various phytochemicals. In this light, screening the antiviral activities of a cocktail of flavonoids would be advantageous in order to prevent viral infections and improve current antiviral therapies.

## 1. Introduction 

**Figure 1:**
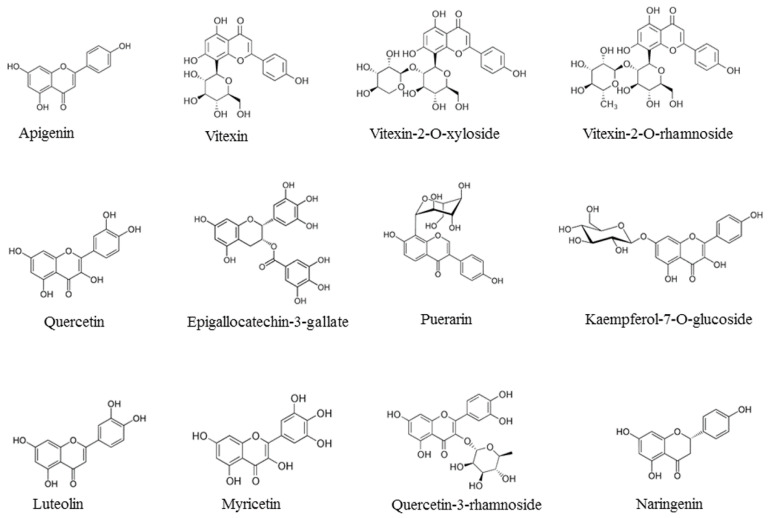
Chemical formulae of representative flavonoids with antiviral activities: apigenin, vitexin, vitexin-2-*O*-xyloside, vitexin-2-*O*-rhamnoside, quercetin, epigallocatechin-3-gallate, puerarin, kaempferol-7-*O*-glucoside, luteolin, myricetin, quercetin-3-rhamnoside, naringenin.

In the past two decades, studies conducted in our laboratory focused on the antioxidants present in vegetable foods and on their capacity to reduce the adverse physiological effects of the oxygen free radicals [1,2]. The research was also aimed at the technologies of food transformation in order to preserve, as much as possible, the antioxidants in the final products [3]. Antioxidants are mainly represented in nature by the liposoluble vitamins E and A, β-carotene, hydro-soluble vitamin C and a wide range of amphipathic molecules, broadly termed phenolic compounds [1]. These compounds are divided into several sub-classes, including phenolic acids and analogues, stilbenes, flavonoids and their analogues. Flavonoids possess important health protective effects, including anti-inflammatory, anticancer and antiviral properties [4,5,6,7,8,9,10]. There are in nature more than 6000 flavonoids, which have been structurally identified and divided in classes: flavones (e.g., apigenin), flavanols (e.g., quercetin), flavins (e.g., epigallocatechin-3-gallate), isoflavones (e.g., genistein) and anthocyanidins (e.g., cyanidin). Flavonoids occur in their free or conjugated form or are often esterified with one or two sugars with *O*-glycosidic or C-glycosidic bonds [11]. In the past decade we purified and studied the biological effects of a group of the flavonoid C-glycosides derived from apigenin, namely vitexin, vitexin-2-*O*-rhamnoside and vitexin-2-*O*-xyloside (Figure 1). 

The interest in the antiviral activity of natural flavonoids has increased in the last decade because of the frequency of viral infections, particularly influenza infections, which affect several million patients annually [12]. While vaccination is the primary strategy for influenza prevention, there are scenarios for which vaccination is not possible and antiviral molecules represent an important sanitary presidium. Synthetic antiviral drugs often show limited efficacy and serious adverse effects [13], whereas herbal extracts, known for their antiviral properties with no or mild side effects, may be a viable alternative for treating various viral diseases [14]. Viruses consist of nucleic acid (DNA or RNA) enclosed in a protein structure, called capsid, which can be surrounded by a lipid membrane named the envelope. The infective unit of the virus is called the virion and this parasite can replicate only inside host cells, hijacking the molecular machinery and controlling DNA replication, RNA transcription and the protein translation processes. Viruses attack the host cells through adsorption to receptors specific for each type of target cells, penetrating through the cell membrane, then the genetic material of the virus is liberated to replicate its own genome, produce new viral proteins and obtain new virions [15]. In 2017, the antiviral effects of various phytochemicals were reviewed by Kapoor et al. [15], taking into consideration many categories of compounds, ranging from flavonoids to saponins and lignans. In the same year, another group published a review focused specifically on the antiviral effects of the six classes of flavonoids [16]. In 2020, an interesting review regarding the methods for delivery of phytochemicals to increase their bioavailability in human tissues has been published [17]. 

In this review, we first summarize flavonoids along with their class and plant sources, with particular attention to apigenin, vitexin and its derivatives (Table 1). We then discuss the antiviral action mechanisms of flavonoids, their combinations with conventional antiviral drugs in multi-target cocktails, and the delivery strategies used to increase their bioavailability and antiviral efficacy.

## 2. Antiviral Effects of Flavonoids

The most common flow chart for studies regarding the antiviral activity of phytochemicals is focused on the immediate testing of the whole natural extract, by dividing the polar from the non-polar constituents. After that, fractions with remarkable activity in in vitro antiviral assays, such as cytophatic effect, neutralization assay, hemagglutination, viral enzyme inhibition, or virion number reduction assay, are further fractionated through chromatographic techniques in order to purify the active phytochemicals. Effective compounds are then used on virus-infected animals or in human clinical trials in order to assess their effective antiviral properties [15]. The complete procedure, when interfaced with chemical libraries, represents the basis for high throughput screening assays [15]. The parameter used for assessing antiviral efficiency of drugs and phytochemicals is represented by 50% Inhibitory Concentrations (IC_50_) or by 50% Effective Concentration (EC_50_). Table 2 shows the main flavonoids studied for antiviral activity along with their investigated mechanisms of action. In the first section, we point out the studies focused on apigenin, vitexin and their derivatives, which were found active against many viruses like human Hepatitis C Virus (HCV), Herpes Simplex Virus-1 (HSV-1), human Hepatitis A and B Viruses (HAV; HBV), rhesus rotavirus (RRV) and influenza virus.

### 2.1. Flavonoids Targeting HCV, HBV and HAV Viruses

The natural extract of *Eclipta alba* (Asteraceae) was shown to be able to inhibit the HCV replicase in a cell culture system, which resulted in reduced HCV RNA titer and translation level of viral proteins [19]. Through bioassay-based fractionations of the natural extract, the authors identified two flavonoid compounds: apigenin and luteolin, which, tested individually, exhibited a dose-dependent inhibition of HCV replicase in vitro [19]. Quercetin, extracted from *Embelia ribes* (Mirsinaceae), exhibited antiviral effects against HCV, exerted through activity inhibition of the viral protease Non-Structural protein 3 (NS3), leading to a decrease in HCV replication [36]. Furthermore, the flavanol quercetagetin was found to inhibit HCV RNA-dependent RNA polymerase (RdRp) through inhibition of RNA binding to the viral polymerase, a mechanism associated with broad genotypic activity against several HCV strains and a high barrier to resistance mechanisms [39]. Luteolin and quercetin showed anti-HCV effects in hepatoma Huh-7 cells transfected with Non-Structural protein 5B (*NS5B*) cloned gene vector, that codifies for the NS5B polymerase of HCV virus [40]. Naringenin and quercetin possess antiviral activity against HCV, but naringenin showed stronger inhibition of virion assembly, whereas quercetin inhibited viral translation by blocking Non-Structural protein 5A (NS5A) and Internal Ribosome Entry Site (IRES)-mediated translation, as well as heat shock protein 70 (HSP70) induction [41]. Bioinformatics tools were also used to study the interaction of various phytochemicals with viral proteins that possess pivotal roles in the production of new virions and in the infection of the host cells. This approach may be a useful premise for deeper investigation regarding flavonoids which have provided interesting evidence of interactions with viral proteins. For instance, naringenin, diosmetin, apigenin and luteolin were all able to bind to the NS5B protein of HCV with higher affinity when compared with the antiviral drug sofosbuvir, inhibiting the activity of this viral enzyme [25]. 

Epigallocatechin-3-gallate (EGCG), the principal tea derived catechin, efficiently inhibited cell culture-derived HBV entry into hepatocellular cell lines, independent of the HBV genotypes, through a mechanism that involves the clathrin-dependent endocytosis of the HBV receptor sodium taurocholate co-transporting polypeptide (NTCP) from the plasma membrane, followed by its protein degradation [26].

The extract obtained from *Erythrina speciosa* (Fabaceae) exerted antiviral effects against HAV-H10 viruses mainly due to vitexin which, isolated from the extract, exhibited an antiviral activity against this virus with EC_50_ = 41.6 ± 7.6 µM [42]. The authors showed that the flavone vitexin can interact with the binding pocket of HAV 3C proteinase, inhibiting this enzyme [42]. 

### 2.2. Antiviral Effects of Flavonoids Against Influenza Viruses 

Vitexin, extracted from the plant *Flos Trollii*, (Caryophyllaceae), exerted its anti-H1N1 influenza virus effects through partially down-regulating Toll-Like Receptor 3 (TLR3) and Toll-Like Receptor 7 (TLR7) pathways and up-regulating Toll-Like Receptor 4 (TLR4) molecular pathway [38]. TLRs are transmembrane glycoproteins, which are privileged targets of several viruses and can be activated by endogenous molecules in the context of inflammation. TLR activation produces pro-inflammatory cytokines through Nuclear factor kappa-light-chain-enhancer of activated B cells (NF-kB) signaling pathway or through Interferon regulatory factor 3 (IRF3) and Interferon regulatory factor 7 (IRF7). Some viruses enter the host cells by binding TLR3, but after their entrance the viruses are able to inhibit cytokine production, thus impairing the immune response. Phytochemicals able to decrease the binding between TLRs and virus particles can slow the infective process. Interestingly, virus infection can lead to an induction of the inflammation process, characterized by excessive production of nitric oxide (NO), Interleukin-1 (IL-1), Interleukin-6 (IL-6) and Tumor Necrosis Factor-α (TNF-α). It was shown that various flavonoids enhance the production of Interferon-β (IFN-β) in order to counteract the viral infections [38]. Baicalin, baicalein, wogonin, chrysin and oroxylin A, isolated from *Scutellaria baicalensis*, showed anti-H1N1 activities, with IC_50_ values of 7.4, 7.5, 2.1, 7.7 and 12.8 μM, respectively, and were all more potent than the conventional antiviral drug oseltamivir phosphate, which had an IC_50_ of 45.6 μM [22]. These flavonoids increased the transcriptional activity of Nuclear factor erythroid 2-related factor 2 (Nrf2), the master regulator of the antioxidant responses, whose activation is related to the antiviral effects of *Scutellaria baicalensis* [22]. The natural extract of *Tetrastigma hemsleyanum* (Vitaceae) contains many flavonoids, including vitexin, vitexin-2-*O*-rhamnoside, isorhamnetin, rutin, kaempferol, astragalin, quercitrin, quercetin and iso-quercetin, which were shown to be able to exert anti-influenza virus activity, with different efficiency, through the reduction of the number of plaques induced by the influenza virus in infected Madin-Darby Canine Kidney (MDCK) cells [21]. Similarly, the flavonoid quercetin-3-rhamnoside, extracted from *Houttuynia cordata* (Saururaceae), exerted anti-influenza A/WS/33 activity reducing virus-mediated cytopathic effects, directly interacting with virus particles [37]. Furthermore, the same authors showed that quercetin-3-rhamnoside exerted anti-influenza virus activity in mice, when used at 6.25 mg/Kg/day for six days after influenza virus infection [45].

### 2.3. Antiviral Properties of Flavonoids Against Dengue and Zika Viruses

Flavonoids Sanggenon O, EGCG and Chamaejasmin were all potentially able to inhibit Dengue virus replication by blocking the Asn-130 glycosylation site of the viral protein Non Structural protein 1 (NS1) [23]. Baicalin, a flavonoid derived from *Scutellaria baicalensis* (Lamiaceae), exhibited viricidal activity against Dengue Virus-2 (DENV-2) extracellular virions with IC_50_ = 19.6 ± 0.2 µM, exerted an anti-adsorption effect with IC_50_ = 40.5 ± 0.4 µM and also inhibited virus replication with IC_50_ = 30.2 ± 0.2 µM [44]. Studies of the antiviral effects of the flavonoids fisetin, quercetagetin, and pinocembrin showed that fisetin can inhibit the replication molecular machineries of Dengue virus and Enterovirus A71 [35]. Furthermore, other antiviral mechanisms of the pinocembrin consist in targeting the molecular machinery used by the Zika virus to replicate its own genome inside the host cells [33]. This flavanone acts on the post-entry processes of Zika virus replication cycle through the inhibition of both viral RNA production and synthesis of envelope proteins [33].

### 2.4. HSV, Respiratory Syncytial Virus (RSV), RRV: Antiviral Activities of Flavonoids

Interestingly, the plant *Moringa oleifera Lam* (Moringaceae) provides a rich and rare combination of several phytochemicals, including the flavonoids quercetin and kaempferol, and its leaves extract can be applied as a prophylactic or therapeutic anti-HSV-1 medicine [29]. The extract obtained from *Moringa oleifera Lam* remarkably reduced the plaque formation induced by wt HSV, thymidine kinase-deficient HSV and phosphonoacetate-resistant HSV strains [29]. Furthermore, the extract obtained from Erythrina speciosa possessed antiviral properties against the HSV-1 virus, mainly due to vitexin which exhibited an antiviral activity with EC_50_ = 80.9 ± 6.2 µM, exerted through the interaction of this flavone with the binding pocket of HSV-1 thymidine kinase [42]. 

Vitexin and luteolin from *Aspalathus linearis* (Fabaceae) showed antiviral activity against RRV with IC_50_ of 129 μM and 116 μM_,_ respectively; interestingly, apigenin-7-*O*-glucoside from *Melissa officinalis* (Labiateae) inhibited viral growth, with an IC_50_ of 150 μM, through the reduction of the number of RRV-induced plaques in infected MA104 cells [20].

Tangeretin and nobiletin, two polymethoxyflavones extracted from *Citrus reticulate* “Chachi”, possess anti-RSV properties. Tangeretin exhibited a dose-dependent inhibition of RSV-induced plaque formation on HEp-2 cells, through inhibition of RSV entry into host cells and viral replication. Furthermore, tangeretin decreased the levels of RSV phosphoprotein (P protein), which is associated with the viral genome to form the holo-nucleocapsid. The extent of the antiviral effect of this phytochemical was comparable to the conventional antiviral drug ribavirin [32].

## 3. Insights into Flavonoid Molecular Targets and Antiviral Synergistic Effects

### 3.1. Influenza Virus Molecular Targets

**Figure 2:**
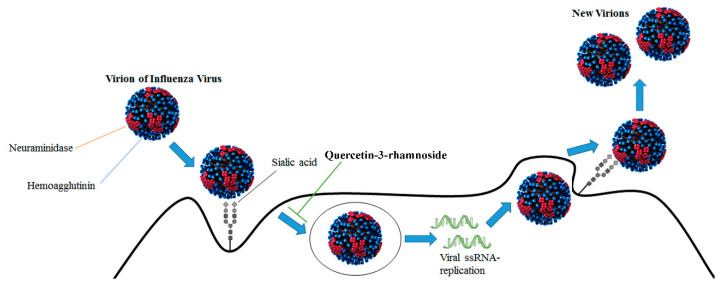
Viral life cycle of Influenza viruses (V Baltimore class: negative ssRNA) and phytochemical quercetin-3-rhamnoside used as virus inhibitor. Virion of influenza virus [46].

The knowledge of the molecular mechanisms of virus infection and phytochemical antiviral actions is fundamental in planning an effective therapeutic approach. The main mechanisms involved in the antiviral effects of phytochemicals are focused on the targeting of viral enzyme activities. Many natural molecules target the catalytic activity of the influenza virus neuraminidase, also called sialidase. This enzyme is a glycoside hydrolase that cleaves the glycosidic linkages of sialic acids (Figure 2).

Various phytochemicals inhibit the activity of viral sialidases, hampering the release of new virions from the infected cells and preventing new infections [47]. Another enzyme with a pivotal role in influenza A virus infection is RdRp, which is composed of three subunits: Polymerase Acidic subunit (PA), Protein Binding 1 subunit (PB1) and Protein Binding 2 subunit (PB2). The enzyme synthesizes viral mRNAs using short capped primers derived from host cellular mRNAs, which are cut by the viral endonuclease. The N-terminal domain of the PA subunit contains a typical endonuclease active site and harbors the RNA/DNA endonuclease activity, which is essential for viral growth [48].

### 3.2. Molecular Targets of HCV and HBV Viruses

**Figure 3:**
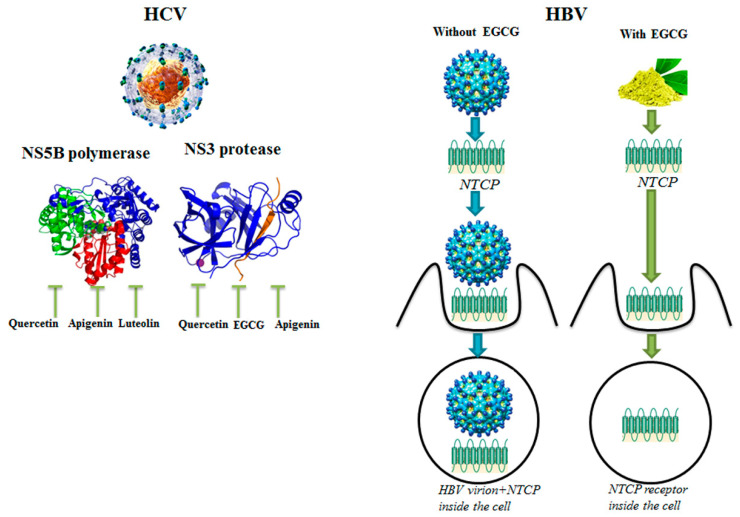
On the left, phytochemicals quercetin, apigenin and luteolin can inhibit the catalytic activity of Hepatitis C Virus (HCV) Non-Structural Protein 5B (NS5B) polymerase, while phytochemicals quercetin, epigallocatechin-3-gallate (EGCG) and apigenin are able to inhibit the activity of HCV NS3 protease. On the right, EGCG induces the internalization of the Hepatitis B Virus (HBV) receptor sodium taurocholate co-transporting polypeptide (NTCP), inhibiting entry of HBV virus in human hepatocytes. HCV virion [49]; HBV virion [50].

Enzymes involved in the HCV virus replication, like the protease NS3 and the polymerase NS5B can also be efficiently inhibited or modulated by phytochemicals (Figure 3). NS3 is a HCV nonstructural protein, which acts as a serine protease. Its N-terminal domain can interact with the viral Non Structural protein 2 (NS2), while the C-terminal domain acts both as helicase and nucleoside triphosphatase. 

The NS5B protein is RdRp with a pivotal role in replicating HCV’s viral RNA by using the viral ssRNA+ as a template to catalyze the polymerization of ribo-nucleoside triphosphates during replication of the viral genome. Interestingly, quercetin, apigenin and luteolin effectively inhibit NS5B polymerase activity [33]. When phytochemicals have been combined among them or with synthetic antiviral drugs, synergistic therapeutic effects were often evidenced. Overall, when synergy occurred, it was often justified by the fact that the different molecules target different steps in the molecular mechanisms of viral infection, and the final antiviral effect results therefore potentiated. Naringenin is a flavanone, which exhibits anti-HCV activity by blocking the assembly of HCV particles [41]. The phytochemical quercetin exerted anti-HCV activity by reducing Internal Ribosome Entry Site- (IRES-)mediated translation and also inhibiting the viral non-structural protein NS5A as well as the protease NS3 [41]. When naringenin and quercetin were used together they suppressed HCV RNA replication and inhibited viral replication to a higher extent when compared with the phytochemicals used individually, thus demonstrating a synergistic effect [41]. Ladanein, a flavone isolated from *Marrubium peregrinum L.* (Lamiaceae), exploited antiviral effects through the inhibition of the post-attachment entry step of HCV virions, with an IC_50_ of 2.5 µM. Ladanein, in combination with cyclosporine, showed a remarkable synergistic antiviral effect against various HCV genotypes [30]. The natural extracts from *Nymphaea alba L*. (Nymphaeaceae), containing iso-quercetin, hyperoside, quercetin, reynoutrin, apigenin and isokaempferide, showed anti-HCV activity, suppressing HCV *NS3* gene expression in the transfected Huh-7 cell line and inhibiting the viral genotype 1a replication. Furthermore, the combination of *Nymphaea alba L.* extract with the conventional drug boceprevir displayed synergistic effects for inhibition of HCV replication in a docking bioinformatics model [28]. An antiviral role of phytochemicals was also linked to the receptors recognized by viruses for their endocytosis, such as the membrane receptor NTCP. This protein has a mass of 56 KDa and is involved in the transport of bile salt molecules, steroid hormones, thyroid hormones and xenobiotics. NTCP is required for the entry in hepatocytes of both HBV and human Hepatitis D Virus (HDV). In fact, the virus-receptor interaction leads to the clathrin-dependent endocytosis of HBV virions, which can replicate inside the host cells [26]. EGCG, used at the dose of 50 μM, induced clathrin-dependent endocytosis of NTCP from the plasma membrane, leading to its degradation and inhibiting the entry of HBV virus into immortalized human primary hepatocytes (Figure 3). 

### 3.3. HIV Protease and Reverse Transcriptase Are Targeted by Flavonoids

**Figure 4:**
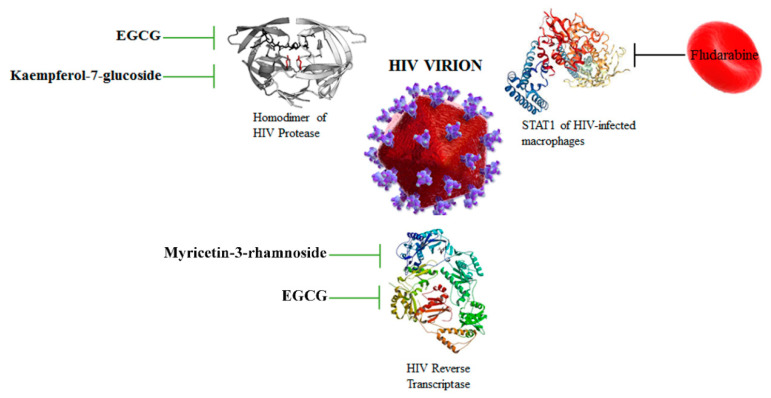
Phytochemicals EGCG and kaempferol-7-glucoside can inhibit the activity of the homodimer of HIV protease, while EGCG and myricetin-3-rhamnoside are able to inhibit the catalytic activity of HIV reverse transcriptase. Various antiviral compounds have been loaded into human red blood cells in order to use these drugs against HIV and HIV-infected cells, like Fludarabine. HIV virion [51]. Signal transducer and activator of transcription 1 (STAT1).

Two HIV enzymes address pivotal roles in virus replication and virion production: HIV reverse transcriptase and the homodimer of HIV protease (Figure 4). HIV reverse transcriptase is an RNA-dependent DNA polymerase (RdDp) and builds ssDNA based on an RNA template in its polymerase active site; the enzyme also cleaves the original RNA template into pieces through its nuclease active site and finally it polymerizes a second DNA strand to form the final dsDNA, which is integrated in the host cell genome. Interestingly, it was shown that EGCG suppressed HIV-1 infection in Hela-CD4-LTR-β-gal cells, with a EC_50_ of 1.6 µM, by acting as a non-nucleoside HIV reverse transcriptase inhibitor [43]. Furthermore, it was demonstrated that the flavonoids myricetin-3-rhamnoside and myricetin-3-(6-rhamnosylgalactoside) possessed antiviral activity in vitro against HIV with EC_50_ of 120 µM and 45 µM, respectively [31]. This antiviral effect was exerted through the inhibition of HIV reverse transcriptase with IC_50_ of 10.6 μM for myricetin-3-rhamnoside and of 13.8 µM for myricetin-3-(6-rhamnosylgalactoside) [31].

HIV protease is a retroviral aspartyl protease, which cleaves newly synthesized viral polyproteins (namely Gag-Pol) at nine cleavage sites to create the mature protein components of the virion. Mature HIV-1 protease exists as a 22 KDa homodimer, each one containing an Asp25 amino acid, which acts as the catalytic residues are able to hydrolyze peptide bonds on the Gag-Pol polyproteins into fully functional viral proteins, like reverse transcriptase and integrase. Kehinde et al. (2019) [27] showed that the phytochemicals kaempferol-7-*O*-glucoside and EGCG were able to interact with HIV-1 protease, showing pronounced structural evidence as potential HIV-1 protease inhibitors (Figure 4). Interestingly, phytochemicals can also be used to reduce the extrusion of antiviral drugs from infected cells. In fact, apigenin, fisetin and luteolin were able to slow down the elimination of the antiviral drugs atazanavir, lopinavir, darunavir and saquinavir, which target the viral protease of HIV-1 [27].

### 3.4. HSV-1 and DENV-2: Decreasing ROS to Counteract Viral Infections

Antiviral activity of flavonoids may be also due to the modulation of host cell enzymes used by the virus to take advantage for infection, such as the enzymes with a pivotal role in the production of Radical Oxygen Species (ROS), utilized to increase the number of virions. Regarding HSV-1, which persists in the host in sensory neurons in latency, the enzyme Nicotinamide Adenine Dinucleotide Phosphate (NADPH) oxidase (NOX) family is a useful source of ROS, which can be used to reactivate the viral infection under oxidative stress conditions [52,53]. Interestingly, delphinidin-3-rutinoside obtained from extracts of *Solanum Melongena L.* (Solanaceae), inhibited HSV-1 replication through the reduction of NOX4 protein levels, when added for 24 h after viral adsorption on Vero cells [24]. The extract obtained from *Veronica Persica Poir* (Plantaginaceae) possessed a dose-dependent inhibitory activity against the herpes simplex virus strains HSV-1 and HSV-2 and a prominent synergistic activity in combination with acyclovir in anti-HSV therapy, exerted through a reduction of the percentage of plaque numbers of both HSV-1 and HSV-2 in the infected cero cells [54]. 

The natural extract of *Disticella elongata* (Bignoniaceae) exhibited antiviral effects against DENV-2 virus and this effect was mainly due to Pectolinarin and acacetin-7-*O*-rutinoside [18]. When the two flavonoids Pectolinarin and acacetin-7-*O*-rutinoside were used in combination, the antiviral effect was eight times more potent against DENV-2 virus (with EC_50_ = 18.3 ± 2.6 µM) than the flavonoid Pectolinarin used alone (with EC_50_ = 138.8 ± 6.1 µM). The selectivity index of the combination (SI = 45) was remarkably higher than the SI of the isolated Pectolinarin (SI = 4.6), indicating that the phytochemical mixture specifically inhibited viral growth, with negligible effects on the vitality of the cells infected by DENV-2 virus. The ethanol extract obtained from the leaves of *Disticella elongata* showed antioxidant activities; therefore, it could detoxify cell damaging free radicals present in DENV-2 viral infections [18].

## 4. Delivery Strategies for Antiviral Drugs and Phytochemicals

The low water solubility and low bioavailability of natural flavonoids represent the major limit for their use in the nutraceutical sector. Many delivery strategies have been used by researchers for increasing flavonoid bioavailability following oral consumption [16], including: micelles, nanoparticles, microspheres, crystals, dendrimers, the Self-Micro-emulsifying Drug Delivery System (SMDDS) and the Self-Nanoemulsifying Drug Delivery systems (SNEDDS), as recently reviewed [17,55]. For instance, it was shown that the catechin and EGCG-loaded chitosan nanoparticles led to a higher rate of intestinal absorption of the two phytochemicals [56]. Interestingly, in chitosan particles the flavonoids maintain their antioxidant activity and can exploit their antioxidant effects in the blood stream [57]. The loading of myricetin into a cationic polymeric nanoparticle carrier with a cationic corona and hydrophobic core was investigated in order to improve the clinical relevance of this natural flavonoid by increasing its solubility [58]. SMDDS has been used to overcome the problem of low bioavailability of hydrophobic molecules as, in the intestinal lumen, the oil-in-water microemulsions containing phytochemicals may be efficiently formed with a consequent increase of the intestinal absorption of the flavonoid [59]. Interestingly, puerarin, an isoflavone isolated from the root of *Pueraria lobata*, exhibited 2.6 fold higher bioavailability when prepared using the SMDDS technique [34]. Furthermore, the synthesis of silver nanoparticles linked with phytochemicals and their use for antimicrobial and antiviral treatments have been described, highlighting the various molecular mechanisms that lead to the phytochemical-mediated inhibition of viral replication [60]. Poly (d,l-Lactide) (PLA) nanoparticles and polymeric micelles contributed to a more sustainable and efficient release of flavonoids characterized by a poor bioavailability, like quercetin and apigenin [61,62]. Quercetin was successfully encapsulated on the most uniformly distributed type of PLA-4 nanoparticle, synthesized using *Lonicera japonica* leaf extract, showing that these nanoparticles allowed a slow release of quercetin [63]. This nanoparticle approach paves the way for encapsulating drugs, small molecules, nutraceuticals and other bioactive ingredients to obtain safer cellular uptake, improved biodistribution, specific targeted delivery and enhancement of the therapeutic antiviral efficacy of encapsulated drugs and phytochemicals [64]. The increase in antiviral efficacy and bioavailability of flavonoids may be attained through their encapsulation into red blood cells (RBCs), as has occurred for other type of antiviral drugs and molecules, such as fludarabine (Figure 4) [65], vincristine and vinblastine [66,67]. The idea of using RBCs as delivery system for flavonoids takes its advantage from the favorable characteristics of these cells. They have a long life-span of about 120 days and have a widespread circulation throughout the body, and hence can be used as drug reservoirs, enabling them to facilitate sustained drug release. Moreover, RBCs protect encapsulated drugs and molecules from degradation; they are completely biodegradable and show no or only minor immunogenic responses. Interestingly, the RBCs were used to determine cellular antioxidant activity of some flavonoids, specifically vitexin, vitexin-2-*O*-xyloside and vitexin-2-*O*-rhamnoside, with the aim of predicting their bioavailability [68]. Moreover, it was demonstrated that flavonoids could have beneficial effects in preventing oxidative damage of erythrocyte membrane [69,70]. Some authors have also evidenced the possibility that human RBCs play a pivotal role in the distribution and bioavailability of circulating flavonoids such as quercetin [71]. Furthermore, it was shown that drug-loaded RBCs can be modified in order to increase their macrophage-mediated phagocytosis by inducing band 3 clustering and opsonization through the complementary and autologous IgGs [72]. 

In future perspective, this approach could be considered in order to possibly improve the antiviral activity of some flavonoids, like baicalin, that was able, like fludarabine [65], to act against HIV-1 chronic infection of human monocytes and macrophages, inhibiting the fusion of HIV virus envelope proteins with these cells [73]. 

Although polymeric nanoparticles, liposomes, dendrimers, micelles and inorganic nanoparticles have been widely accepted as drug delivery systems, they show toxicity and a short lifetime, thus making them relatively disadvantageous when compared with natural cell carriers, such as RBCs. For this reason, some authors in recent years have tried to mimic the erythrocyte cell membrane to produce biocompatible nanocarriers in order to decrease their toxicity and to prolong their survival in blood circulation [74,75,76]. RBCs, which are biodegradable and non-immunogenic, can be used as a valuable carrier system with a lifespan that is remarkably prolonged and controllable when compared to synthetic carriers. Several approaches have been developed to load peptides, drugs and molecules into RBCs or to attach these agents onto RBCs’ outer membrane surface by either chemical or physical methods [77] and the possibility of loading drugs into autologous RBCs prior to their transfusion into patients has been studied in small animal models and primates, as well as in clinical studies of human patients [78,79,80,81]. The topic of phytochemical encapsulation in RBCs remains open for further studies, but we believe that flavonoid derivatives and nanoparticles able to bind flavonoids could be successfully considered for this application in the near future.

## 5. Therapeutic Potential of Antiviral Dietary Flavonoids 

Diet and life-style play important roles in the defense against the attacks of viruses. The relationship between diet and the immune system involves the microbiota, i.e., the ecological community of commensals and potentially pathogenic microbes and symbionts that live in the gut [82]. The diet modulates the microbiota composition, leading to an increase or a decrease in immune defenses [82]. The Mediterranean Diet (and in general diets rich in fruits, vegetables and herbs) maintains gut microbiota homeostasis and provides protection against microbes and viruses [83]. The cross-talk between microbiota and immunity is supported by the Aryl Hydrocarbon Receptor (AhR), which is ubiquitous, but mainly present in the cytoplasm of immune cells [84]. It was demonstrated that AhR binds different ligands, namely metabolites, pollutants and pathogenic molecules, and after this interaction it translocates into the nucleus, where it induces specific transcription programs and modulates the defensive functions of both T and B cells, dendritic cells and monocytes [84]. Interestingly, it was shown that flavonoids can bind AhR [84]. On this basis, people eating vegetables, all of which contain flavonoids to a different extent, would strengthen their immune system [83]. This strengthening is a general effect that occurs with many nutrients, but there are more specific antiviral effects attributable to the flavonoids treated in this review. Flavonoids, like apigenin, vitexin, quercetin, rutin and naringenin, show a wide range of antiviral effects (Table 2), because they are able to target different pathways of viral infections. It is therefore possible to increase the chances of blocking viral replication using mixtures of flavonoids with synergistic antiviral effects, because of the pleiotropic effects of their combination. 

An important question that arises is focused on the reasonable concentration range of flavonoids that should be used for an effective antiviral therapy, which is hard to be reached by diet alone. Recent experiments performed in in vivo studies demonstrated the protective efficacy of various flavonoids, tested in the range 10–50 mg/Kg body weight per day, in newborn mice challenged with a lethal dose of the enterovirus 71 [85]. Interestingly, apigenin (50 mg/Kg), luteolin (10 mg/Kg) and quercetin (10 mg/Kg) conferred survival protection of 88.9%, 91.7% and 50.1%, respectively, from the lethal enterovirus 71 infection; moreover, isorhamnetin provided the highest survival protection of 100% at a dose of 10 mg/Kg. The authors hypothesized that the differences in concentrations are due to different times of absorption and renal clearance of these flavonoids [85]. The flavanol isorhamnetin was studied also by Dayem et al. [86], who showed that oral administration of this flavonoid at 1 mg/Kg/day for five days in mice infected with the influenza A virus significantly decreased lung virus titer by two-fold, increased the survival rate (which ranged from 70% to 80%) and decreased mice body weight loss by 25%. These authors hypothesized that the methyl group of the B ring of isorhamnetin may contribute to its strong antiviral potency against influenza A virus [86]. Guo et al. [87] focused their in vivo studies on the flavone wogonin, showing that, in human HBV-transgenic mice, this flavonoid administered once a day at 7, 14 and 28 mg/Kg reduced plasma HBsAg level and immunohistological staining of the liver confirming the HBsAg reduction exerted by wogonin [87]. The potentiality of flavonoid bioactivity in vivo depends on the extent of their absorption after ingestion and their ability for distribution in various target tissues. In this light, Liu et al. [88] developed a quercetin-loaded cationic nanostructure lipid carrier (Q-CNLC), which increased the in vivo bioavailability of this flavonoid. This quercetin-nanostructure complex benefits from its strong interactions with negatively charged intestinal mucosa, which could increase its residence in the gastrointestinal tract. Moreover, the authors showed an entrapment efficiency of quercetin of 89.3% and a slower release of this flavonoid from the Q-CLNC, when compared with the release profile of a simple quercetin solution, indicating that Q-CLNC exhibited a sustained and controlled release of this flavanol, in order to maintain its effective therapeutic concentration [88]. In addition, the same authors performed in vivo tissue distribution studies in C57BL/6J mice, comparing treatment with 25 mg/kg of orally administered quercetin solution with the administration of 25 mg/Kg of Q-CNLC, showing that the relative quercetin uptake from Q-CNLC was 1.57 fold higher in lungs, 1.51 fold higher in liver and 1.68 fold higher in kidneys. On the contrary, the relative quercetin uptake from Q-CNLC was lower in spleen, heart and brain, when compared with the quercetin solution [88]. These results indicate that the most suitable delivery strategy should be chosen in order to target a specific organ with a particular flavonoid-nanostructure complex for future clinical applications. Furthermore, the safety of the used phytochemical should also be considered, as has already been done for hydroxytyrosol, which is quickly absorbed and eliminated by the kidneys in either free or conjugated forms. Hydroxytyrosol has been considered safe at 50 mg/Kg/day by the European Food safety Authority (EFSA) panel [89]. In this light, we think that, for a 70 Kg person, a flavonoid range between 0.5–3.0 g/day should be proposed. These values are close to the daily polyphenol intake in humans, calculated in various diet surveys [90], such as the Supplementation en Vitamines et Mineraux Antioxydants (SU.VI.MAX) study, which ranked the polyphenol intake at 1.19 ± 0.51 g/day [91]. Accordingly, in another dietary intervention trial aimed at improving cognition in older adults, a total of 1.04 g/day of flavonoids was applied [92]. Indeed, based on these results, we think that 0.5–3.0 g/day of flavonoids could be a reasonable concentration range in order to start preliminary experiments, focused on assessing the in vivo antiviral effects of flavonoids.

Concerning the combination of flavonoids in an antiviral cocktail, each phytochemical may be used initially at a concentration of about 0.5 g/day with the aim of reaching an intake of 3 g/day of antiviral flavonoids. In the case of antiviral synergistic effects [5,6] or increase of the absorption of one specific flavonoid exerted by other phytochemicals [93], the individual flavonoid concentrations can be modulated, according to the extent of the effect. It should also be emphasized that a significant antiviral effect is linked to the type of flavonoid mixture, the delivery system used, the pharmacokinetic pattern, the number of targets involved and the number of required daily doses. Once these aspects have been defined, the more suitable regimen of administration consists of starting with the lowest effective concentration for a fixed time and proceeding with increasing doses of the antiviral flavonoids. Monitoring the markers of antiviral efficacy and any side effects should also be considered in order to evaluate the risks-benefits pattern.

## 6. Conclusions and Future Perspectives

Overall, our review shows that many flavonoids exhibit antiviral activity and could offer a promising alternative for prevention of and therapy for viral infections. Flavonoids are present in many vegetables and the first protection for the immune system resides in the ability to seek foods rich in bioactive nutrients. Education programs for a healthy diet should be implemented during the outbreaks of viral infections [94]. In fact, a diet rich in vegetables activates the AhR in the gut for maintenance of microbiota homeostasis, which in turn regulates the immune system. In the critical periods of viral infections, oral dietary supplementation with nutraceutical preparations based on combinations of flavonoids can be useful in order to inhibit different steps of the viral infective cycle. Molecular mechanisms underlying the antiviral effects of flavonoids, herein described, mainly focus on the inhibition of viral enzyme activities: neuraminidases, DNA/RNA polymerases and proteases. Therefore, a cocktail of flavonoids, selected for their efficacy in the inhibition of different viral enzymes, could be associated with elevated immune response and offer a promising option for antiviral therapies. This option acquires great importance considering that the viral genome frequently mutates, due to the lack of proof-reading activity of most of the viral polymerases. These mutations could hamper the efficacy of antiviral synthetic drugs. On the contrary, antiviral flavonoids, as well as the combination of synthetic antiviral drugs with flavonoids, would enhance therapeutic strategies by targeting the multiple signaling pathways involved in the viral infections [95]. The active concentration of the flavonoids should be investigated, considering the pharmacokinetic studies available in the literature and the synergistic effects of the specific flavonoid combinations [15,16,17].

The scarce intestinal absorption and bioavailability of flavonoids, when given through food or in pills, may be enhanced by the use of new drug delivery strategies [96]. In fact, since flavonoids have some drawbacks after oral administration such as low stability, bioavailability and bio-efficacy, researchers are developing biocompatible nanomaterial synthesis as novel delivery systems (including nanospheres, nano-capsules, micro and nano-emulsions, micelles, solid lipid nanoparticles and capsules), for overcoming the delivery challenges of flavonoids in the biomedical sector. Phytochemical-nanomaterial complexes can represent innovative drug delivery strategies (alongside those already known) for new antiviral therapies against the seven Baltimore virus classes [97]. 

Interestingly, three patents regarding the antiviral effects of flavonoids (US 7,998,937; EP1245230; US 6,399,654) have been already assigned to the Korea Research Institute of Bioscience and Biotechnology and Advanced Life Sciences Inc. [35]. 

However, an important step that must be achieved is to obtain the authorization of novel food including flavonoids with antiviral properties, following regulation EU 2015/2283 [98]. Nowadays, this authorization has been given only to hydroxytyrosol [89] and few other nutrients. If the antiviral efficacy and safety of flavonoids and their mixtures can be clearly demonstrated in vivo, it would be possible to obtain European Food Safety Authority (EFSA) authorization of novel foods, in order to provide new natural tools for preventing and facing outbreaks of viral infections. Indeed, when flavonoids are administered through nano-sized delivery systems, they show increased stability and bioavailability with an enhanced and prolonged activity. However, the in vivo behavior and the antiviral actions of these nano-delivery systems are still under experimental evaluation.

## Figures and Tables

**Table 1:** Classification and sources of the flavonoids described in this Review.

Flavonoid	Class	Sources	Ref.
Acacetin-7-*O*-rutinoside	Flavone	*Disticella elongata*	[18]
Apigenin	Flavone	*Eclipta alba*	[19]
Apigenin-7-*O*-glucoside	Flavone	*Melissa officinalis*	[20]
Astragalin	Flavanol	*Tetrastigma hemsleyanum*	[21]
Baicalein	Flavone	*Scutellaria baicalensis*	[22]
Baicalin	Flavone	*Scutellaria baicalensis*	[22]
Chamaejasmin	Flavanone	*Enkleia siamensis*	[23]
Chrysin	Flavone	*Scutellaria baicalensis*	[22]
Delphinidin-3-rutinoside	Anthocyanidin	*Solanum Melongena*	[24]
Diosmetin	Flavone	*Pistacia chinensis*	[25]
Epigallocatechin-3-gallate	Flavin	*Camellia sinensis*	[26]
Fisetin	Flavanol	*Carica papaya*	[27]
Hyperoside	Flavanol	*Nymphaea alba*	[28]
Isokaempferide	Flavanol	*Nymphaea alba*	[28]
Isorhamnetin	Flavanol	*Tetrastigma hemsleyanum*	[21]
Isoquercetin	Flavanol	*Tetrastigma hemsleyanum*	[21]
Kaempferol	Flavanol	*Moringa oleifera Lam*	[29]
Kaempferol-7-*O*-glucoside	Flavanol	*Carica papaya*	[27]
Ladanein	Flavone	*Marrubium peregrinum*	[30]
Luteolin	Flavone	*Eclipta alba*	[19]
Myricetin	Flavanol	*Marcetia taxifolia*	[31]
Myricetin-3-rhamnoside	Flavanol	*Marcetia taxifolia*	[31]
Myricetin-3-(6-rhamnosylgalactoside)	Flavanol	*Marcetia taxifolia*	[31]
Naringenin	Flavanone	*Citrus grandis Osbeck*	[25]
Nobiletin	Flavone	*Citrus reticulate*	[32]
Oroxylin A	Flavone	*Scutellaria baicalensis*	[22]
Pectolinarin	Flavone	*Disticella elongata*	[18]
Pinocembrin	Flavanone	*Pinus sibirica*	[33]
Puerarin	Isoflavone	*Pueraria lobata*	[34]
Quercetagetin	Flavanol	*Tagetes mandonii*	[35]
Quercetin	Flavanol	*Embelia ribes*	[36]
Quercetin-3-rhamnoside	Flavanol	*Houttuynia cordata*	[37]
Quercitrin	Flavanol	*Tetrastigma hemsleyanum*	[21]
Reynoutrin	Flavanol	*Nymphaea alba*	[28]
Rutin	Flavanol	*Tetrastigma hemsleyanum*	[21]
Sanggenon O	Flavanone	*Morus alba*	[23]
Tangeretin	Flavone	*Citrus reticulate*	[32]
Vitexin	Flavone	*Flos Trollii*	[38]
Vitexin-2-*O*-rhamnoside	Flavone	*Tetrastigma hemsleyanum*	[21]
Vitexin-2-*O*-xyloside	Flavone	*Beta vulgaris cicla*	[10]
Wogonin	Flavone	*Scutellaria baicalensis*	[22]

**Table 2:** Molecular mechanisms and targets of antiviral activities of flavonoids reported in this review.

Flavonoids	Inhibited Virus	Targets	Molecular Mechanisms	Ref.
Apigenin, Luteolin	HCV	HCV replicase	Inhibition of HCV replication	[19]
Vitexin, Apigenin-7-*O*-glucoside,	Rhesus Rotavirus	Rotavirus virions	Inhibition of viral replication	[20]
Vitexin	H1N1 influenza	TLR3, TLR4,	Decrease of inflammatory injury,	[38]
		TLR7 pathways	Increase of IFN-β levels	
Vitexin	HSV-1 and HAV	HSV-1 virions,	Inhibition of viral replication	[42]
		HAV virions		
Apigenin, Isoquercetin, Quercetin	HCV	NS3 protease	Inhibition of HCV replication	[18]
Quercetin-3-rhamnoside	Influenza A/WS/33	Influenza virions	Inhibition of virus infection	[37]
EGCG	HIV	Reverse transcriptase	Inhibition of HIV replication	[43]
Myricetin-3-rhamnoside	HIV	Reverse transcriptase	Inhibition of HIV replication	[31]
Quercetin, Catechin, Naringenin	HCV	NS5A, HSP70,	Inhibition of viral translation,	[41]
		HCV virions	Inhibition of virion assembly	
Delphinidin-3-rutinoside	HSV-1	NOX4	Inhibition of HSV-1 replication	[24]
EGCG	HBV	NTCP receptor	Inhibition of HBV entry into cells	[26]
Quercetin	HCV	NS3 protease	Inhibition of HCV replication,	[36]
			Inhibition of virion production	
Luteolin, Quercetin	HCV	NS5B polymerase	Inhibition of HCV replication	[33]
EGCG,	Dengue	NS1	Inhibition of NS1 glycosylation	[23]
Sanggenon O, Chamaejasmin				
Baicalin	Dengue Virus-2 (DENV-2)	DENV-2 virions	Inhibition of viral replication,	[40]
			Viricidal activity	
Baicalin, Baicalein	H1N1 influenza	Nrf2	Inhibition of viral replication	[44]
Luteolin	HCV	NS5B polymerase	Inhibition of HCV replication	[40]
Naringenin, Quercetin	HCV	Envelope 2 protein,	Inhibition of virion assembly,	[41]
		NS5A, NS3	Inhibition of HCV entry into cells	
Tangeretin, Nobiletin	RSV	Phosphoprotein P	Inhibition of viral replication,	[32]
			Inhibition of RSV entry into cells	
Kaempferol-7-*O*-glucoside, EGCG	HIV	HIV protease	Inhibition of virion production	[27]
Quercetagetin	HCV	NS5B polymerase	Inhibition of RNA binding to NS5B	[39]
Pinocembrin	Zika	Viral RNAs	Envelope protein synthesis inhibition	[33]

## Abbreviations

AhR	Aryl Hydrocarbon Receptor
DENV-2	Dengue Virus-2
EC_50_	50% Effective Concentration
EFSA	European Food Safety Authority
EGCG	Epigallocatechin-3-gallate
HAV	Human Hepatitis A Virus
HBV	Human Hepatitis B Virus
HCV	Human Hepatitis C Virus
HDV	Human Hepatitis D virus
HIV	Human Immunodeficiency Virus
HSP70	Heat Shock Protein 70
HSV-1	Herpes Simplex Virus type-1
IC_50_	50% Inhibitory Concentration
IFN-β	Interferon-β
IL-1	Interleukin-1
IL-6	Interleukin-6
IRES	Internal Ribosome Entry Site
IRF3	Interferon regulatory factor 3
IRF7	Interferon regulatory factor 7
MDCK	Madin-Darby Canine Kidney
NADPH	Nicotinamide Adenine Dinucleotide Phosphate
NF-kB	Nuclear factor kappa-light-chain-enhancer of activated B cells
NO	Nitric Oxide
NOX4	NADPH Oxidase 4
Nrf2	Nuclear factor erythroid 2-related factor 2
NS1	Non Structural protein 1
NS2	Non Structural protein 2
NS3	Non Structural protein 3
NS5A	Non Structural protein 5A
NS5B	Non Structural protein 5B
NTCP	sodium taurocholate cotransporting polypeptide
P protein	RSV phosphoprotein
PA	Polymerase Acidic subunit
PB1	Protein Binding 1 subunit
PB2	Protein Binding 2 subunit
PLA	Poly (d,l-Lactide)
Q-CNLC	Quercetin-loaded Cationic Nanostructure Lipid Carrier
RBCs	Red Blood Cells
RdDp	RNA-dependent DNA polymerase
RdRp	RNA-dependent RNA polymerase
ROS	Radical Oxygen Species
RRV	Rhesus rotavirus
RSV	Respiratory Syncytial Virus
SMDDS	Self-Micro-emulsifying Drug Delivery System
SNEDDS	Self-Nanoemulsifying Drug Delivery System
STAT1	Signal transducer and activator of transcription 1
SU.VI.MAX	Supplementation en Vitamines et Mineraux Antioxydants
TLR3	Toll-Like Receptor 3
TLR4	Toll-Like Receptor 4
TLR7	Toll-Like Receptor 7
TNF-α	Tumor Necrosis Factor-α

## References

[1] Ninfali P., Bacchiocca M. (2003). Polyphenols and antioxidant capacity of vegetables under fresh and frozen conditions. J. Agric. Food Chem..

[2] Ninfali P., Aluigi G., Bacchiocca M., Magnani M. (2001). Antioxidant capacity of Extra-Virgin Olive Oils. JOACS.

[3] Ninfali P., Mea G., Giorgini S., Rocchi M., Bacchiocca M. (2005). Antioxidant capacity of vegetables, spices and dressings relevant to nutrition. Br. J. Nutr..

[4] Lee E.R., Kang G.H., Cho S.G. (2007). Effect of flavonoids on human health: Old subjects but new challenges. Recent Pat. Biotechnol..

[5] Papi A., Farabegoli F., Iori R., Orlandi M., De Nicola G.R., Bagatta M., Angelino D., Gennari L., Ninfali P. (2013). Vitexin 2-*O*-xyloside, raphasatin and (-) eepigallocatechin-3-gallate synergistically affect cell growth and apoptosis of colon cancer cells. Food Chem..

[6] Farabegoli F., Scarpa E.S., Frai A., Serafini G., Papi A., Spisni E., Antonini E., Benedetti S., Ninfali P. (2017). Betalains increase vitexin-2-*O*-xyloside cytotoxicity in Caco-2 cancer cells. Food Chem..

[7] Scarpa E.S., Antonini E., Palma F., Mari M., Ninfali P. (2018). Antiproliferative activity of vitexin-2-*O*-xyloside and avenanthramides on CaCo-2 and HepG2 cancer cells occurs through apoptosis induction and reduction of pro-survival mechanisms. Eur. J. Nutr..

[8] Scarpa E.S., Mari M., Antonini E., Palma F., Ninfali P. (2018). Natural and synthetic avenathramides activate caspases 2,8,3 and downregulate hTERT, MDR1 and COX-2 genes in CaCo-2 and Hep3B cancer cells. Food Funct..

[9] Antonini E., Iori R., Ninfali P., Scarpa E.S. (2018). A Combination of Moringin and Avenanthramide 2f Inhibits the Proliferation of Hep3B Liver Cancer Cells Inducing Intrinsic and Extrinsic Apoptosis. Nutr. Cancer.

[10] Ninfali P., Antonini E., Frati A., Scarpa E.-S. (2017). C-Glycosyl Flavonoids from Beta vulgaris Cicla and Betalains from Beta vulgais rubra: Antioxidant, Anticancer, Antiinflammatory Activities—A Review. Phytother. Res..

[11] Ninfali P., Bacchiocca M., Antonelli A., Biagiotti E., Di Gioacchino A.M., Piccoli G., Stocchi V., Brandi G. (2007). Characterization and biological activity of the main flaonoids from Swiss Chard (Beta vulgaris subspecies cycla). Phytomedicine.

[12] Moscona A. (2005). Oseltamivir resistance-disabling our influenza defenses. N. Engl. J. Med..

[13] Hostettmann K., Marston A. (2002). Twenty years of research into medicinal plants: Results and perspectives. Phytochem. Rev..

[14] Ni L., Zhou L., Zhou M., Zhao J., Wang D.W. (2020). Combination of western medicine and Chinese traditional patent medicine in treating a family case of COVID-19 in Wuhan. Front. Med..

[15] Kapoor R., Sharma B., Kanwar S.S. (2017). Antiviral Phytochemicals: An Overview. Biochem. Physiol..

[16] Zakaryan H., Arabyan E., Oo A., Zandi K. (2017). Flavonoids: Promising natural compounds against viral infections. Arch. Virol..

[17] Ben-Shabat S., Yarmolinsky L., Porat D., Dahan A. (2020). Antiviral effects of phytochemicals from medicinal plants: Applications and drug delivery strategies. Drug Deliv. Transl. Res..

[18] Simoes L.R., Maciel G.M., Brandao G.C., Kroon E.G., Castilho R.O., Oliveira A.B. (2011). Antiviral activity of Disticella elongata (Vahl) Urb. (Bignoniaceae), a potentially useful source of anti-dengue drugs from the state of Minas Gerais, Brazil. Lett. Appl. Microbiol..

[19] Manvar D., Mishra M., Kumar S., Pandey V.N. (2012). Identification and evaluation of anti hepatitis C virus phytochemicals from Eclipta alba. J. Ethnopharmacol..

[20] Knipping K., Garssen J., van’t Land B. (2012). An evaluation of the inhibitory effects against rotavirus infection of edible plant extracts. Virol. J..

[21] Ding F., Liu J., Du R., Yu Q., Gong L., Jiang H., Rong R. (2019). Qualitative and Quantitative Analysis for the Chemical Constituents of Tetrastigma hemsleyanum Diels et Gilg Using Ultra-High Performance Liquid Chromatography/Hybrid Quadrupole—Orbitrap Mass Spectrometry and Preliminary Screening for Anti-Influenza Virus Components. Evid. Based Complement. Alternat. Med..

[22] Ji S., Li R., Wang Q., Miao W.J., Li Z.W., Si L.L., Qiao X., Yu S.W., Zhou D.M., Ye M. (2015). Anti-H1N1 virus, cytotoxic and Nrf2 activation activities of chemical constituents from Scutellaria baicalensis. J. Ethnopharmacol..

[23] Qamar M.T., Mumtaz A., Naseem R., Ali A., Fatima T., Jabbar T., Ahmad Z., Ashfaq U.A. (2014). Molecular Docking Based Screening of Plant Flavonoids as Dengue NS1 inhibitors. Bioinformation.

[24] Di Sotto A., Di Giacomo S., Amatore D., Locatelli M., Vitalone A., Toniolo C., Rotino G.L., Lo Scalzo R., Palamara A.T., Marcocci M.E. (2018). A Polyphenol Rich Extract from Solanum melongena L. DR2 Peel Exhibits Antioxidant Properties and Anti-Herpes Simplex Virus Type 1 Activity In Vitro. Molecules.

[25] Mirza M.U., Ghori N.U., Ikram N., Adil A.R., Manzoor S. (2015). Pharmacoinformatics approach for investigation of alternative potential hepatitis C virus nonstructural protein 5B inhibitors. Drug Des. Devel. Ther..

[26] Huang H.C., Tao M.H., Hung T.M., Chen J.C., Lin Z.J., Huang C. (2014). (-)-Epigallocatechin-3-gallate inhibits entry of hepatitis B virus into hepatocytes. Antivir. Res..

[27] Kehinde I., Ramharack P., Nlooto M., Gordon M. (2019). The pharmacokinetic properties of HIV-1 protease inhibitors: A computational perspective on herbal phytochemicals. Heliyon.

[28] Rehman S., Ashfaq U.A., Ijaz B., Riazuddin S. (2018). Anti-hepatitis C virus activity and synergistic effect of Nymphaea alba extracts and bioactive constituents in liver infected cells. Microb. Pathog..

[29] Anwar F., Latif S., Ashraf M., Gilani A.H. (2007). Moringa oleifera: A food plant with multiple medicinal uses. Phytother. Res..

[30] Haid S., Novodomskà A., Gentzsch J., Grethe C., Geuenich S., Bankwitz D., Chhatwal P., Jannack B., Hennebelle T., Bailleul F. (2012). A Plant-Derived Flavonoid Inhibits Entry of All HCV Genotypes Into Human Hepatocytes. Gastroenterology.

[31] Ortega J.T., Suarez A.I., Serrano M.L., Baptista J., Pujol F.H., Rangel H.R. (2017). The role of the glycosyl moiety of myricetin derivatives in anti-HIV-1 activity in vitro. AIDS Res. Ther..

[32] Xu J.J., Wu X., Li M.M., Li G.Q., Yang Y.T., Luo H.J., Huang W.H., Chung H.Y., Ye W.C., Wang G.C. (2014). Antiviral activity of polymethoxylated flavones from Guangchenpi, the edible and medicinal pericarps of Citrus reticulata “Chachi”. J. Agric. Food Chem..

[33] Lee L.J., Loe M.W., Lee R.C., Chu J.J. (2019). Antiviral activity of pinocembrin against Zika virus replication. Antivir. Res..

[34] Zhang Y., Wang R., Wu J., Shen Q. (2012). Characterization and evaluation of self-microemulsifying sustained-release pellet formulation of puerarin for oral delivery. Int. J. Pharm..

[35] Ghildiyal R., Prakash V., Chaudhary V.K., Gupta V., Gabrani R., Swany M.K. (2020). Phytochemicals as Antiviral Agents: Recent Updates. Plant-Derived Bioactives.

[36] Bachmetov L., Gal-Tanamy M., Shapira A., Vorobeychik M., Giterman-Galam T., Sathiyamoorthy P., Golan-Goldhirsh A., Benhar I., Tur-Kaspa R., Zemel R. (2012). Suppression of hepatitis C virus by the flavonoid quercetin is mediated by inhibition of NS3 protease activity. J. Viral Hepat..

[37] Choi H.J., Song J.H., Park K.S., Kwon D.H. (2009). Inhibitory effects of quercetin 3-rhamnoside on influenza A virus replication. Eur. J. Pharm. Sci..

[38] Shi D., Chen M., Liu L., Wang Q., Liu S., Wang L., Wang R. (2020). Anti-influenza A virus mechanism of three representative compounds from Flos Trollii via TLRs signaling pathways. J. Ethnopharmacol..

[39] Ahmed-Belkacem A., Guichou J.F., Brillet R., Ahnou N., Hernandez E., Pallier C., Pawlotsky J.M. (2014). Inhibition of RNA binding to hepatitis C virus RNA-dependent RNA polymerase: A new mechanism for antiviral intervention. Nucleic Acid Res..

[40] Rehman S., Ijaz B., Fatima N., Muhammad S.A., Riazuddin S. (2016). Therapeutic potential of Taraxacum officinale against HCV NS5B polymerase: In-vitro and In silico study. Biomed. Pharmacother..

[41] Khachatoorian R., Arumugaswani V., Raychauduri S., Yeh G.K., Maloney E.M., Wang J., Dasgupta A., French S.W. (2012). Divergent antiviral effects of bioflavonoids on the hepatitis C virus life cycle. Virology.

[42] Fahmy N.M., Al-Sayed E., Moghannem S., Azam F., El-Shazly M., Singab A.N. (2020). Breaking Down the Barriers to a Natural Antiviral Agent: Antiviral Activity and Molecular Docking of Erythrina speciosa Extract, Fractions, and the Major Compound. Chem. Biodivers..

[43] Li S., Hattori T., Kodama E.N. (2011). Epigallocatechin gallate inhibits the HIV reverse transcription step. Antivir. Chem. Chemother..

[44] Moghaddam E., Teoh B.T., Sam S.S., Lani R., Hassandarvish P., Chik Z., Yueh A., Abubakar S., Zandi K. (2014). Baicalin, a metabolite of baicalein with antiviral activity against dengue virus. Sci. Rep..

[45] Choi H.J., Song J.H., Kwon D.H. (2012). Quercetin-3-rhamnoside exerts antiinfluenza A virus activity in mice. Phytother. Res..

[46] Virology Blog: About Viruses and Viral Disease. https://www.virology.ws/2014/12/10/how-influenza-virus-infection-might-lead-to-gastrointestinal-symptoms/.

[47] Glanz V.Y., Myasoedova V.A., Grechko A.V., Orekhov A.N. (2018). Inhibition of sialidase activity as a therapeutic approach. Drug Des. Dev. Ther..

[48] Iwai Y., Murakami K., Gomi Y., Hashimoto T., Asakawa Y., Okuno Y., Ishikawa T., Hatakeyama D., Echigo N., Kuzuhara T. (2011). Anti-influenza activity of marchantins, macrocyclic bisbibenxyls contained in liverworts. PLoS ONE.

[49] Hepatitis C Virus. https://en.wikipedia.org/wiki/Hepatitis_C_virus.

[50] HBV-GLUE: A Sequence Data Resource for Hepatitis B Virus. http://hbv-glue.cvr.gla.ac.uk/#/home.

[51] Corriere Nazionale: I 5 Virus Che Spaventano La Scienza. https://www.corrierenazionale.it/2020/02/09/i-5-virus-che-spaventano-la-scienza/.

[52] De Chiara G., Marcocci M.E., Sgarbanti R., Civitelli L., Ripoli C., Piacentini R., Garaci E., Grassi C., Palamara A.T. (2012). Infectious agents and neurodegeneration. Mol. Neurobiol..

[53] Amatore D., Sgarbanti R., Aquilano K., Baldelli S., Limongi D., Civitelli L., Nencioni L., Garaci E., Ciriolo M.R., Palamara A.T. (2015). Influenza virus replication in lung epithelial cells depends on redox-sensitive pathways activated by NOX4-derived ROS. Cell. Microbiol..

[54] Sharifi-Rad J., Iriti M., Setzer W.N., Sharifi-Rad M., Roointan A., Salehi B. (2018). Antiviral activity of Veronica Persica Poir. on herpes virus infection. Cell. Mol. Biol..

[55] Lembo D., Cavalli R. (2010). Nanoparticulate delivery systems for antiviral drugs. Antivir. Chem. Chemother..

[56] Dube A., Nicolazzo J.A., Larson I. (2010). Chitosan nanoparticles enhance the intestinal absorption of the green tea catechins (+)-catechin and (-)-epigallocatechin gallate. Eur. J. Pharm. Sci..

[57] Casettari L., Gennari L., Angelino D., Ninfali P., Castagnino E. (2012). ORAC of chitosan and its derivatives. Food Hydrocoll..

[58] Sims K.R., He B., Koo H., Benoit D.S.W. (2020). Electrostatic Interactions Enable Nanoparticle Delivery of the Flavonoid Myricetin. ACS Omega.

[59] Kang B.K., Lee J.S., Chon S.K., Jeong S.Y., Yuk S.H., Khang G., Lee H.B., Cho S.H. (2004). Development of self-microemulsifying drug delivery system (SMEDDS) for oral bioavailability enhancement of simvastatin in beagle dogs. Int. J. Pharm..

[60] Koduru J.R., Kailasa S.K., Bhamore J.R., Kim K.H., Dutta T., Vellingiri K. (2018). Phytochemical-assisted synthetic approaches for silver nanoparticles antimicrobial applications: A review. Adv. Colloid Interface Sci..

[61] Kumari A., Yadav S.K., Pakade Y.B., Singh B., Yadav S.C. (2010). Development of biodegradble nanoparticles for delivery of quercetin. Colloids Surf. B Biointerfaces.

[62] Zhai Y., Guo S., Liu C., Yang C., Dou J., Li L., Zhai G. (2013). Preparation and in vitro evaluation of apigenin-loaded polymeric micelles. Colloids Surf. A.

[63] Kumari A., Kumar V., Yadav S.K. (2012). Plant extract synthesized PLA nanoparticles for controlled and sustained release of quercetin: A green approach. PLoS ONE.

[64] Martínez-Ballesta M.C., Gil-Izquierdo Á., García-Viguera C., Domínguez-Perles R. (2018). Nanoparticles and Controlled Delivery for Bioactive Compounds: Outlining Challenges for New “Smart-Foods” for Health. Foods.

[65] Magnani M., Balestra E., Fraternale A., Aquaro S., Paiardini M., Cervasi B., Casabianca A., Garaci E., Perno C.F. (2003). Drug-loaded red blood cell-mediated clearance of HIV-1 macrophage reservoir by selective inhibition of STAT1 expression. J. Leukoc. Biol..

[66] Jeewantha H.M.A., Slivkin A.I. (2018). The terpene-indole alkaloids loaded erythrocytes as a drug carrier: Design and assessment. Clin. Pharmacol..

[67] Trineeva O.V., Khalahakun A.D. (2019). Study of desorbtion and exemption of terpeno-indole alkaloids of vinkristin and vinblastin from erythrocitary cell carriers. Drug Dev. Regist..

[68] Blasa M., Angelino D., Gennari L., Ninfali P. (2011). The cellular antioxidant activity in red blood cells (CAA-RBC): A new approach to bioavailability and synergy of phytochemicals and botanical extracts. Food Chem..

[69] Asgary S., Naderi G.H., Askari N. (2005). Protective effect of flavonoids against red blood cell hemolysis by free radicals. Exp. Clin. Cardiol..

[70] Hou L., Zhou B., Yang L., Liu Z.L. (2004). Inhibition of free radical initiated peroxidation of human erythrocyte ghosts by flavonols and their glycosides. Org. Biomol. Chem..

[71] Fiorani M., Accorsi A., Cantoni O. (2003). Human Red Blood Cell sas a Natural Flavonoid Reservoir. Free Radic. Res..

[72] Serafini S., Rossi L., Antonelli A., Fraternale A., Cerasi A., Crinelli R., Chiarantini L., Schiavano G.F., Magnani M. (2004). Drug delivery through phagocytosis of red blood cells. Transfus. Med. Hemotherapy.

[73] Lalani S., Poh C.L. (2020). Flavonoids as Antiviral Agents for Enterovirus A71 (EV-A71). Viruses.

[74] Xia Q., Zhang Y., Li Z., Hou X., Feng N. (2019). Red blood cell membrane-camouflaged nanoparticles: A novel drug delivery system for antitumor application. Acta Pharm. Sin. B.

[75] Doshi N., Zahr A.S., Bhaskar S., Lahann J., Mitragotri S. (2009). Red blood cell-mimicking synthetic biomaterial particles. Proc. Natl. Acad. Sci. USA.

[76] Merkel T.J., Jones S.W., Herlihy K.P., Kersey F.R., Shields A., Napier M., Luft J.C., Wu H., Zamboni W.C., Wang A. (2011). Using mechanobiological mimicry of red blood cells to extend circulation times of hydrogel microparticles. Proc. Natl. Acad. Sci. USA.

[77] Antonelli A., Magnani M. (2016). Engineering erythrocytes for the modulation of drugs’ and contrasting agents’ pharmacokinetics and biodistribution. Adv. Drug Deliv. Rev..

[78] Magnani M., Serafini S., Fraternale A., Antonelli A., Biagiotti S., Pierigè F., Sfara C., Rossi L., Nalwa H.S. (2011). Red blood cell-based delivery of drugs and nanomaterials for therapeutic and diagnostic applications. Encyclopedia of Nanoscience and Nanotechnology.

[79] Villa C.H., Pan D.C., Zaitsev S., Cines D.B., Siegel D.L., Muzykantov V.R. (2015). Delivery of drugs bound to erythrocytes: New avenues for an old intravascular carrier. Ther. Deliv..

[80] Flower R., Peiretti E., Magnani M., Rossi L., Serafini S., Gryczynski Z., Gryczynski I. (2008). Observation of erythrocyte dynamics in the retinal capillaries and choriocapillaris using ICG-loaded erythrocyte ghost cells. Investig. Ophthalmol. Vis. Sci..

[81] Villa C.H., Cines D.B., Siegel D.L., Muzykantov V. (2017). Erythrocytes as carriers for drug delivery in blood transfusion and beyond. Transfus. Med. Rev..

[82] Sirisinha S. (2016). The potential impact of gut microbiota on your health: Current status and future challenges. Asian Pac. J. Allergy Immunol..

[83] Gibson A., Edgar J.D., Neville C.E., Gilchrist S., McKinley M.C., Patterson C., Young I.S., Woodside J.V. (2012). Effect of fruit and vegetable consumption on immune function in older people: A randomized controlled trial. Am. J. Clin. Nutr..

[84] Koper J.E.B., Loonen L.M.P., Wells J.M., Troise A.D., Capuano E., Fogliano V. (2019). Polyphenols and Tryptophan Metabolites Activate the Aryl Hydrocarbon receptor in an in vitro Model of Colonic Fermentation. Mol. Nutr. Food Res..

[85] Dai W., Bi J., Li F., Wang S., Huang X., Meng X., Sun B., Wang D., Kong W., Jiang C. (2019). Antiviral Efficacy of Flavonoids Against Enterovirus 71 Infection in Vitro and in Newborn Mice. Viruses.

[86] Dayem A.A., Choi H.Y., Kim Y.B., Cho S.G. (2015). Antiviral effect of methylated flavonol isorhamnetin against influenza. PLoS ONE.

[87] Guo Q., Zhao L., You Q., Yang Y., Gu H., Song G., Lu N., Xin J. (2007). Anti-hepatitis B virus activity of wogonin in vitro and in vivo. Antivir. Res..

[88] Liu L., Tang Y., Gao C., Li Y., Chen S., Xiong T., Li J., Du M., Gong Z., Chen H. (2014). Characterization and biodistribution in vivo of quercetin-loaded cationic nanostructured lipid carriers. Colloids Surf. B Biointerfaces.

[89] EC 2017 (2017). EC Regulation 258/97. Safety of hydroxytyrosol as a novel food pursuant to Regulation (EC) No 258/97. EFSA J..

[90] Del Bo C., Bernardi S., Marino M., Porrini M., Tucci M., Guglielmetti S., Cherubini A., Carrieri B., Kirkup B., Kroon P. (2019). Systematic Review on Polyphenol Intake and Health Outcomes: Is There Sufficient Evidence to Define a Health-Promoting Polyphenol-Rich Dietary Pattern?. Nutrients.

[91] Perez-Jimenez J., Fezeu L., Touvier M., Arnault N., Manach C., Hercberg S., Galan P., Scalbert A. (2011). Dietary Intake of 337 Polyphenols in French Adults. Am. J. Clin. Nutr..

[92] Brickman A.M., Khan U.A., Provenzano F.A., Yeung L.K., Suzuki W., Schroeter H., Wall M., Sloan R.P., Small S.A. (2014). Enhancing Dentate Gyrus Function With Dietary Flavanols Improves Cognition in Older Adults. Nat. Neurosci..

[93] Ferri P., Angelino D., Gennari L., Benedetti S., Ambrogini P., Del Grande P., Ninfali P. (2015). Enhancement of Flavonoid Ability to Cross the Blood-Brain Barrier of Rats by Co-Administration With α-tocopherol. Food Funct..

[94] Galanakis C.M. (2020). The Food Systems in the Era of the Coronavirus (COVID-19) Pandemic Crisis. Foods.

[95] Sanjuan R., Nebot M.R., Chirico N., Mansky L.M., Belshaw R. (2010). Viral mutation rates. J. Virol..

[96] Bilia A.R., Isacchi B., Righeschi C., Guccione C., Bergonzi M.C. (2014). Flavonoids loaded in Nanocarriers: An Opportunity to Increase Oral Bioavailability and Bioefficacy. Food Nutr. Sci..

[97] Cojocaru F.D., Botezat D., Gardikiotis I., Uritu C.M., Dodi G., Trandafir L., Rezus C., Rezus E., Tamba B.I., Mihai C.T. (2020). Nanomaterials Designed for Antiviral Drug Delivery Transport across Biological Barriers. Pharmaceutics.

[98] EU 2015 (2015). Commission Regulation (EU) 2015/2283. Regulation (EU) 2015/2283 of the European Parliament and of the Council of 25 November 2015 on novel foods, amending Regulation (EU) No 1169/2011 of the European Parliament and of the Council and repealing Regulation (EC) No 258/97 of the European Parliament and of the Council and Commission Regulation (EC) No 1852/2001. OJL.

